# Dr. Bryant Sloat Fassett

**Published:** 1908-05

**Authors:** 


Dr. Bryant Sloat Fassett, of New York, formerly of Elmira, N. Y., died in Roosevelt Hospital, New York, March 24, 1908, of acute nephritis after an illness of twelve days, aged 29 years. He was a son of Hon. J. Sloat Fassett, M.C., and a graduate of the College of Physicians and Surgeons, New York, 1903; formerly surgeon to outdoor department of Roosevelt Hospital and to Vanderbilt Clinic; a member of the medical societies, State and County of New York, and of the Elmira Academy of Medicine.

